# Door-to-Skin Time in Patient Undergoing Emergency Trauma Craniotomy

**DOI:** 10.21315/mjms2023.30.4.7

**Published:** 2023-08-24

**Authors:** Kumarappan Chockalingam, Noor Azman A Rahman, Zamzuri Idris, Sharon Casilda Theophilus, Jafri Malin Abdullah, Abdul Rahman Izaini Ghani, Aisyah Ali

**Affiliations:** 1 Department of Neurosurgery, Hospital Sultanah Aminah Johor Bahru, Johor, Malaysia; 2 Department of Neurosciences, School of Medical Sciences, Universiti Sains Malaysia, Kelantan, Malaysia; 3 Brain and Behaviour Cluster, School of Medical Sciences, Universiti Sains Malaysia, Kelantan, Malaysia; 4 Clinical Research Centre, Hospital Sultan Ismail, Johor, Malaysia

**Keywords:** traumatic brain injury, emergency trauma craniotomy, door-to-skin time, door-to-CT time, organised trauma system

## Abstract

**Background:**

Traumatic brain injury (TBI) is the third leading cause of death and disability worldwide in 2020. For patients with TBI with significant intracranial bleeds, urgent surgical intervention remains the mainstay treatment. This study aims to evaluate the time to definite surgical intervention since admission and its association with patient outcomes in a neurosurgery referral centre in Malaysia.

**Methods:**

This retrospective study was conducted at Hospital Sultanah Aminah Johor Bahru from 1 January 2019 to 31 December 2019. All patients with TBI requiring urgent craniotomy were identified from the operating theatre registry, and the required data were extracted from their clinical notes, including the Glasgow Outcome Score (GCS) at discharge and 6 months later. Logistic regression was performed to identify the factors associated with poor outcomes.

**Results:**

A total of 154 patients were included in this study. The median door-to-skin time was 605 (interquartile range = 494–766) min. At discharge, 105 patients (68.2%) had poor outcomes. At the 6-month follow-up, only 58 patients (37.7%) remained to have poor outcomes. Simple logistic regression showed that polytrauma, hypotensive episode, ventilation, severe TBI, and the door-to-skin time were significantly associated with poor outcomes. After adjustments for the clinical characteristics in the analysis, the likelihood of having poor outcomes for every minute delay in the door-to-skin time increased at discharge (adjusted odds ratio [AOR] = 1.005; 95% confidence interval [CI] = 1.002–1.008) and the 6-month follow-up (AOR = 1.008; 95% CI = 1.005–1.011).

**Conclusion:**

The door-to-skin time is directly proportional to poor outcomes in patients with TBI. Concerted efforts from all parties involved in trauma care are essential in eliminating delays in surgical interventions and improving outcomes.

## Introduction

According to the World Health Organization (WHO), traumatic brain injury (TBI) is the third leading cause of death and disability in 2020 (1). This condition places a significant burden on healthcare especially in developing countries, such as Malaysia. Urgent neurosurgical intervention has remained the only viable treatment option for patients with a significant intracranial bleed or mass effect.

The landmark studies by Mendelow et al. (2) and Seelig et al. (3) are often quoted to emphasise the need for urgent surgical evacuation for extradural and subdural haemorrhage. Mendelow et al. (2) found that delayed evacuation of extradural haemorrhage increased morbidity and mortality; particularly, > 2 h delays were found to be unacceptable. Seelig et al. (3) reported that patients with traumatic subdural haemorrhage who underwent surgery within and after 4 h of injury had mortality rates of 30% and 90%, respectively. Although it is advocated that patients should be subjected to surgical evacuation of the mass lesion as soon as possible, no time cut-off is indicated in the guidelines for surgical management for TBI by the Brain Trauma Foundation (4).

Similarly, in Malaysia, there has been no consensus on how soon patients with TBI should undergo surgery. Although all efforts are taken to ensure that patients receive timely surgical intervention, there are inadvertent delays in their management. To date, no studies have evaluated the performance of neurosurgery referral centres with regard to emergency trauma craniotomy delays in Malaysia. This study aims to determine the time from first healthcare facility arrival to definitive surgical intervention and its association with patient outcomes.

## Methods

### Research Design

This retrospective study was conducted in a single regional neurosurgery centre to evaluate the performance in terms of emergency trauma craniotomy delays. Convenience sampling was used in this study. All patients who underwent emergency trauma craniotomy or craniectomy at Hospital Sultanah Aminah Johor Bahru (HSAJB) from 1 January 2019 to 31 December 2019 were included in accordance with the inclusion and exclusion criteria. The inclusion criteria were as follows: TBI, admission to HSAJB, abnormal brain computed tomography (CT) findings (intra-axial lesions: contusion, traumatic intracranial haemorrhage, extradural haemorrhage, subdural haemorrhage and diffuse axonal injury), and need for urgent neurosurgical intervention (craniotomy or craniectomy). The exclusion criteria were as follows: initial medical management but later requirement for surgery for clinical or radiological deterioration, craniectomy for elevated intracranial pressure (ICP), surgery without intracranial mass lesion (compound or depressed fractures) and missing or incomplete data.

### Research Location and Duration

This study was conducted at HSAJB, which is the first trauma centre to be established in Malaysia. It is the tertiary referral centre for the southern region of peninsular Malaysia, which encompasses a wide range of subspecialties, including neurosurgery and trauma services. Located in the state of Johor in Malaysia, the Neurosurgery Department of HSAJB is the only government neurosurgical centre in the southern region. It is equipped with two operative theatres, one neuro high dependency unit with 10 beds and two neurosurgery wards with 30 beds. It serves the entire state of Johor, covering a wide geographical area of 19,166 km^2^ with an estimated population of about 3.76 million (5). This study was conducted from 1 January 2019 to 31 December 2019.

### Data Collection and Variable Definition

A list of patients who underwent emergency craniotomy or craniectomy was obtained from the operating theatre (OT) registry at HSAJB and their respective clinical records were traced from the record office and clinic notes. Those who met the inclusion and exclusion criteria were filtered. The demographic data and required clinical data were extracted from the clinical notes and filled into the data collection sheet. The Glasgow Coma Scale (GCS) score and pupillary size and reactivity upon arrival in the primary hospital and upon review of the neurosurgical team were noted. Any reductions in the GCS score or changes in the pupillary size or reactivity between hospital arrival and neurosurgery review were considered clinical deterioration. Further, patients with TBI severe enough to require admission were considered polytraumatised. When there were multiple intracranial bleeds on CT, the CT findings were classified on the basis of the lesion requiring surgical intervention.

The patients were classified according to four referral patterns: i) patients who arrived directly at HSAJB; ii) patients referred from specialist hospitals with CT scanners; iii) patients referred from district hospitals to HSAJB for CT and iv) patients transferred from secondary hospitals for CT prior to referral to HSAJB.

The following times were collected: i) time of injury (time when patients sustained TBI, when available); ii) time of arrival at initial hospital (door time); iii) time of CT imaging (CT time); iv) time of neurosurgical team review (review time); v) time of case posting to the anaesthesiology team (booking time); vi) time of general anaesthesia induction in the OT (OT time) and vii) time of skin incision (skin time). For patients referred from other hospitals, the time of arrival at HSAJB (arrival time) was also noted. For patients who underwent CT and received consultation with the neurosurgical team prior to transfer, the times of referral (referral time) and reply (reply time) by email were noted. The door time was determined as the arrival time registered at the first emergency department (ED); CT time: time appearing on the first CT film; review time: time documented by the on-call neurosurgical team when cases were reviewed at HSAJB (routinely documented in our practice); booking time: time when cases were posted for surgery (routinely documented); OT time: time of general anaesthesia induction in the OT; and skin time: time recorded in the OT register as the time of skin incision. We then calculated the delay between the door and CT times, between the CT and review times, between the review and booking times, between the booking and OT times, between the OT and skin times, and between the door and skin times. For patients referred from another hospital, we also calculated the reply and transfer times. The reason for delays was duly noted when available.

The Glasgow Outcome Score (GOS) at discharge from our hospital and during outpatient clinic follow-up at 6 months was collected. A score of 1 indicates death; 2 indicates vegetative state; 3 indicates severe disability (conscious but dependent); 4 indicates moderate disability (independent but with some limitation in terms of work or social life); and 5 indicates good recovery (can resume normal occupation and social activities). In this study, the outcome was divided into two groups: good, with a GOS of 4 or 5, and poor, with a GOS of 1–3.

### Statistical Analysis

Data were entered, cleaned and analysed using the Statistical Package for the Social Sciences version 22.0. Means and standard deviations were used to describe the patient characteristics categorised as continuous data with a normal distribution and medians and interquartile ranges (IQRs) categorised as continuous data with a skewed distribution. Frequencies and percentages were used to describe the patient characteristics categorised as categorical data. The time interval, referral pattern, TBI severity, brain CT finding, polytrauma, ventilation and hypotensive episode were compared using Mood’s median test. Logistic regression was applied to identify the association between the factors and poor outcomes. A *P*-value of < 0.05 was considered statistically significant.

## Results

### Demographic and Clinical Characteristics

A total of 226 patients underwent either emergency trauma craniotomy or craniectomy at HSAJB during the study period. Of them, 72 patients were excluded owing to the following reasons: 34 patients (47.2%), either delayed clinical or radiological deterioration; 18 patients (25.0%), no mass lesion on CT scan; 11 patients (15.3%), delayed surgery after ICP monitoring; and 9 patients (12.5%), incomplete or unavailable data. Ultimately, 154 patients were included in the study. The baseline demographic and clinical characteristics of the included patients are summarised in Table 1. The mean age was 29.7 years old, with the youngest patient being 5 years old and the oldest patient being 72 years old. The most common mechanism of injury was motor vehicle accident in 123 patients (79.9%), followed by falls in 25 patients (16.2%). During neurosurgery review, 58 patients (37.7%) were ventilated and 74 patients (48.1%) had clinically deteriorated since hospital arrival. Extradural haemorrhage (47.4%) and subdural haemorrhage (43.5%) were the two most common indications for emergency trauma craniotomies.

### Referral Patterns

Of the 154 patients, only 28 patients (18.2%) had directly arrived at HSAJB post-trauma, while the remaining 126 patients (81.8%) were transferred to HSAJB from other healthcare facilities. Most patients (*n* = 97, 63.0%) were referred from hospitals with CT scanners. Twenty-four patients (15.6%) were transferred from hospitals without CT scanners to HSAJB for diagnostic CT and later underwent surgery. Five patients (3.2%) had dual transfers: they initially arrived at a hospital without a CT scanner and had to be transferred to another hospital for imaging prior to transfer to HSAJB for definitive surgery.

### Time Interval

The time intervals were divided according to the following referral patterns: direct admission to HSAJB, hospital without CT scanners, hospital with CT scanners, and dual transfer (Table 2). From the time of neurosurgery review at HSAJB, the same chronology was followed in all patients.

### Direct Admission to HSAJB

A total of 28 patients were directly admitted to HSAJB. The median door-to-CT time was 149 min. Thirteen patients (8.4%) presented with a GCS score of < 13, of whom only four patients (23.1%) managed to undergo brain CT within 2 h. The commonest documented cause of delay in performing CT was unavailability of staff or equipment (*n* = 8, 28.6%) and clinical instability for transfer to the radiology suite (*n* = 6, 21.43%).

The median CT-to-neurosurgery review time was 105 min. This time interval was further divided into the CT-to-neurosurgery referral time and neurosurgery referral-to-neurosurgery review time. The median CT-to-neurosurgery referral time was 72 (IQR = 47–90) min, while the median neurosurgery referral-to-review time was 20 (IQR = 10–38) min. The CT-to-neurosurgery review time was prolonged owing to delay in reviewing the CT films by the ED team (*n* = 6, 21.43%), the neurosurgical team attending to other emergency cases (*n* = 3, 10.71%), and difficulty contacting the on-call neurosurgical team (*n* = 2, 7.14%).

### Hospital without CT Scanners

The following three district hospitals within 50 km radius sent patients to HSAJB for imaging: i) Hospital Kulai; ii) Hospital Pontian and iii) Hospital Kota Tinggi. The median door-to-CT time of the patients transferred from these hospitals (*n* = 24) was 267 min. Twenty-two patients (91.7%) presented a GCS score of < 15, but none of them underwent CT within 2 h. The median transfer time to HSAJB was 213 (IQR = 169–243) min. The median time from arrival at HSAJB to brain CT was 40 (IQR = 31–65) min. The commonest cause of delay in performing CT in these patients was unavailability of ambulance or personnel for patient transfer (*n* = 11, 45.83%) and clinical instability for transfer as well as the need for further stabilisation (*n* = 4, 16.67%).

### Hospital with CT Scanners

The following four feeder hospitals transferred 97 patients to HSAJB for surgical intervention: i) Hospital Sultan Ismail; ii) Hospital Muar; iii) Hospital Batu Pahat and iv) Hospital Segamat. The median door-to-CT time in these patients was relatively shorter (103 [25–681] min) than that in the patients admitted directly to HSAJB. Thirty-three of the fifty-three patients (62.3%) presenting a GCS score of < 13 underwent CT within 2 h. Two patients had a door-to-CT time of > 12 h, as they underwent exploratory laparotomy and had to be stabilised before undergoing brain CT. In this subgroup of patients, the CT-to-neurosurgery review time was further divided into the CT-to-neurosurgery teleconsultation time, neurosurgery teleconsultation-to-neurosurgery reply time, neurosurgery reply-to-arrival at HSAJB time (transfer time), and arrival at HSAJB-to-neurosurgery review time. The median CT-to-neurosurgery teleconsultation time was 122 (IQR = 83–174) min, while the median neurosurgery teleconsultation-to-neurosurgery reply time was 26 (IQR = 10–54) min. The usual cause of delay in performing neurosurgery consultation was delay in surgical team review at the primary hospitals (*n* = 10, 10.31%). The commonest cause of delay in replying to the teleconsultation was inadequate clinical information (*n* = 8, 8.25%), incomplete or incorrect images (*n* = 3, 3.10%) and revision of the management plan (*n* = 2, 2.06%).

Of the five hospitals with CT scanners, only one is situated within 17 km from HSAJB; the remaining hospitals are located between 100 km and 170 km away from HSAJB. The median transfer time among the patients transported to HSAJB was 166 (IQR = 122–222 min). The cause of delay in transfer was unavailability of ambulance or personnel (*n* = 26, 26.80%) and clinical instability for patient transfer (*n* = 13, 13.40%). The median arrival at HSJAB-to-neurosurgery review time was 18 (IQR = 8–30) min, the shortest among the groups.

### Dual Transfer

Patients from Hospital Tangkak and Hospital Mersing are routinely transferred to Hospital Batu Pahat and Hospital Sultan Ismail, respectively, for CT prior to referral to HSAJB. When CT machines break down, imaging is outsourced to a nearby private hospital. Herein, only five patients who had dual transfers underwent emergency trauma craniotomy at HSAJB. The median door-to-CT time was the longest in these patients: 416 (IQR = 298–459) min. The median CT-to-neurosurgery review time was 363 (IQR = 314–380) min. There was no documented reason for delay in these patients.

### Neurosurgery Review-to-Skin Time

The median neurosurgery review-to-booking time was 20 (IQR = 15–35) min. The commonest cause of delay in booking for surgery was the need for clinical reassessment after withdrawal of sedation (*n* = 13, 8.44%), need for repeated brain CT prior to decision-making (*n* = 11, 7.14%), and need to wait for consent from family members (*n* = 3, 3.90%).

The median booking-to-OT time was 90 (IQR = 61–130) min. The shortest booking-to-OT time was 20 min, while the longest booking-to-OT time was 485 min. Only five patients managed to arrive in the OT within 30 min of booking, of whom three had mild TBI, and two had moderate TBI. None of the patients with severe TBI arrived in the OT within 30 min. There was no significant difference in the median booking-to-OT time between the patients with mild, moderate, and severe TBI (*χ*^2^ = 3.733, *dF* = 2, *P* = 0.155). The reason for delay in OT transfer included the busyness of the OT with other emergency cases and inability to mobilise another OT (*n* = 22, 14.29%), the lack of personnel or equipment for transfer to the OT (*n* = 8, 5.19%), issues in postoperative placement (*n* = 5, 3.24%), and deterioration or the need for stabilisation (*n* = 3, 1.95%).

The median OT-to-skin time was 62 (IQR = 18–173) min. The prolonged time taken from patient arrival in the OT to skin incision was attributed to the difficulty of anaesthetic procedures (i.e. intubation and establishment of the arterial line and central venous access; *n* = 16, 10.39%), need for further stabilisation prior to initiation of surgery (*n* = 4, 2.60%), and need for emergency laparotomy prior to neurosurgery (*n* = 3, 1.95%).

### Door-to-Skin Time

The median door-to-skin time was 605 (IQR = 494–766) min. Only one patient (0.66%) underwent surgery within 4 h of arrival in the hospital, while 49 patients (31.82%) had to wait for > 12 h from the time of arrival to undergo surgery. The factors affecting the door-to-skin time are described in Table 3. In Mood’s median test, the door-to-skin time was significantly associated with the referral patterns (*P* < 0.001), polytrauma (*P* = 0.007) and hypotensive episode (*P* = 0.009).

### Clinical Factors Associated with Patient Outcomes

Among the 154 patients, the in-hospital mortality rate was 10.39%. At discharge, 102 patients (66.23%) had poor outcomes. In the simple logistic regression analysis, we found that polytrauma, hypotensive episode, ventilation, severe TBI and the door-to-skin time were all significantly associated with poor outcomes at discharge (Tables 4 and 5). However, in the multiple logistic regression analysis, only ventilation (*P* < 0.001) and the door-to-skin time (*P* < 0.001) remained significantly associated with poor outcomes. Thus, ventilation and the door-to-skin time were the factors associated with poor outcomes regardless of the existence of polytrauma, hypotensive episode or severe TBI. This model fitted with a Hosmer and Lemeshow *P*-value of 0.633 and Nagelkerke *R*^2^ value of 0.424. The adjusted odds ratio for the door-to-skin time was 1.005 with a 95% confidence interval (CI) of 1.002, 1.008. Hence, for every minute delay in the door-to-skin time, the likelihood of having poor outcomes at discharge increased by 1.005 times.

At the 6-month follow-up, 58 patients (37.66%) remained to have poor outcomes, including three patients who died, yielding a total mortality rate of 12.34% (*n* = 19). Similar to the poor outcomes at discharge, the poor outcomes at follow-up were significantly associated with polytrauma, hypotensive episode, ventilation, severe TBI, and the door-to-skin time in the simple logistic regression analysis (Tables 4 and 5). However, only the door-to-skin time remained significantly associated with poor outcomes in the multiple logistic regression analysis (*P* < 0.001). Hence, regardless of the clinical characteristics, every minute delay in the door-to-skin time led to a 1.008 (95% CI = 1.005, 1.011)-fold increase in having poor outcomes at 6 months.

## Discussion

In a meta-analysis conducted in 2014, the annual incidence of TBI among all ages was 295 per 100,000 people worldwide (6). Low-to-middle-income countries have been reported to have a threefold larger number of cases of TBI than high-income countries (7). Furthermore, Southeast Asian and Western Pacific regions have the highest burden of the condition. While falls are the commonest cause of TBI in high-income countries, road traffic accidents account for most cases in low-to-middle-income countries (7). This finding is consistent with our data in which the mechanism of injury was motor vehicle accidents in 79.9% of the cases. According to the Department of Statistics of Malaysia, road accidents were the fourth leading cause of death in 2018, accounting for 3.7% of all deaths in Malaysia, and were the commonest cause of death within the age group of 15 years old–40 years old (8). Accordingly, TBI is a major healthcare problem both worldwide and in Malaysia.

TBI is dynamic in nature and consists of primary and secondary injuries. Primary brain injury occurs at the moment of impact and causes either focal or diffuse axonal damage (9). Secondary brain injury occurs after the initial insult in the form of hypoxia, hypovolaemia, and cerebral oedema. While bleeding from the damaged vessels is due to primary brain injury, the resultant extradural or subdural bleeding that accumulates over time is considered part of the secondary brain injury complex (10). It is important to distinguish between the two entities, as primary brain injury is not reversible, while secondary brain injury is preventable and, to a certain extent, reversible if appropriately managed. Most patients with TBI could be managed without surgery. According to the National Trauma Database in the United States, only 3.6% of patients with TBI will require neurosurgical intervention (11).

The phrase ‘Time is brain!’ was originally coined by a neurologist to emphasise that the outcomes of patients with stroke are time critical. The longer the treatment is delayed, the worse the outcomes (2, 3). The landmark studies by Mendelow et al. (2) and Seelig et al. (3) formed the fundamental basis for recommending early surgical intervention for TBI. Many other studies followed suit and drew similar conclusions (11–14) stressing the importance of timely surgical intervention. Our study is in line with these studies and showed that with every minute delay in the door-to-skin time, there was an increase in the odds of having poorer outcomes.

The accessibility to neurotrauma services is rather limited in developing countries, such as Malaysia, and accordingly, delays in reaching neurosurgical care is common. To the best of our knowledge, our study is the first to evaluate the performance of a regional neurosurgery referral centre in Malaysia in handling acute neurotrauma requiring urgent surgical intervention. Therefore, it is unfortunate that we are unable to compare our time intervals with those of other hospitals within this region. A few similar studies have been conducted in various other high- and low-to-middle-income countries, resulting in wide variations in the door-to-skin time. In developed countries such as the United States, the median time between ED admission to neurosurgical intervention is 133 min (15), while according to the neurotrauma database, the median time from injury to craniotomy is 195 min (16). In Canada, the median ED-to-‘knife’ time is 150 min (17), while in Europe Royal London Hospital, the median injury-to-medical care time is 13 min, and the median injury-to-neurosurgery time is 177 min (18). In a level 1 trauma centre in Thailand, the mean time from ED arrival to skin incision is 160 min (19) for traumatic intracranial subdural haematomas. However, notably, accessibility to neurotrauma service is not evenly distributed even in developed countries. Even in the United States, the average time from rural injury to initial care is 1.2 h, and it takes 5 h to reach a level 1 trauma centre (20). In Auckland, the mean time from the scene of injury to neurosurgery is 3 h 50 min, while the mean time from other hospitals to arrival at Auckland City Hospital is > 4 h (21). Nevertheless, these figures seem far-fetched when compared with our findings wherein the median time to direct admission was 459 min.

For most patients with head injury, CT of the head and neck is the imaging modality of choice for detecting intracranial lesions. The indication for CT in patients with TBI has been described in multiple different guidelines, with the Canadian CT Head Rule and New Orleans Criteria being the two most commonly quoted in the literature. According to the Malaysian guidelines on early management of head injury, one indication for an immediate scan (within 1 h–2 h) is a GCS score of < 13 on initial management. In this study, 69 patients had a GCS score of < 13 on initial review; however, only 37 patients (53.6%) managed to undergo CT within 2 h from arrival in the ED. Notably, the door-to-CT time at HSAJB, being a trauma centre, was longer than that in other hospitals equipped with CT scanners. The probable cause for this finding is the physical distance to the CT machine, which is located in a radiology suite away from the ED at HSAJB. In the literature, the door-to-CT time ranges from 45.6 min to 2 h (19, 22, 23). Simple interventions, including education sessions for the multidisciplinary team and junior doctors and distribution of information sheets on prevailing guidelines, have been shown to reduce the mean time to scan from 73 to 55 min (23). In another study involving patients with acute stroke, the door-to-CT time of within 20 min increased from 47% to 74% when the CT scanner was relocated within the ED (24). Alternatively, patients with TBI should be prioritised for a CT scan without delay by closely coordinating with the radiology department.

We also realised that it was impossible for the three district hospitals within 50 km from HSJAB to adhere to the guidelines to perform immediate CT within 2 h. Although the actual transfer time for the patients was not long, most delays occurred at initiation and arrangements for transfer to another centre. The delays were predominantly caused by the lack of staff or equipment for patient transfer. The local clinical practice guideline on head injury in adults advocates that patients with suspected TBI should be preferably transferred from the scene of injury directly to a centre with resources necessary to resuscitate, investigate, and manage patients with polytrauma wherein TBI can be managed in its entirety (25). Although this will be ideal, it will not be possible to accept all patients with TBI to our centre for investigation and management, as it will strain our already limited resources. The Brain Trauma Foundation recommends that patients with severe TBI should be directly transported to facilities with immediate availability of CT scan and prompt neurosurgical care (26). Furthermore, the emphasis must be placed on the need for an organised trauma care system with a protocol to assist decision-making regarding transfer destination. We concur that there is a subset of patients who will benefit from direct transfer from the scene of injury to HSAJB, bypassing the local district hospital; however, further groundwork will be necessary to determine its feasibility.

In most centres, the ED team triages, reviews or resuscitates patients with TBI and performs CT scan if indicated. After brain CT is performed, the ED team refers patients to the in-hospital surgical team in the absence of a neurosurgical team (except at HSAJB). Subsequently, the surgical team reviews patients and refers them to the on-call neurosurgical team at HSAJB. Teleconsultation occurs via phone call and imaging results are conveyed via email. When referral information is incomplete or unclear, further clarification is obtained. Finally, the decision to take over is discussed with the on-call neurosurgeon and conveyed to the surgical team to make transfer arrangements. Furthermore, in the event of polytrauma, which is common in patients with TBI, multiple other specialities (e.g. general surgery or orthopaedic) are notified at the receiving end. In this study, we found that the management of TBI is complex such that it results in delays especially at the referring hospital. Empowering the ED team in peripheral hospitals to directly refer to the on-call neurosurgical team bypassing the surgical team especially in urgent cases may reduce unnecessary delays in referral. A standardised neurotrauma referral form could help the referring team from missing vital information necessary to make decisions, which can prevent the need for repeated clarifications. At the receiving end, referral should be dealt with by neurosurgical personnel competent to make decisions on the need to transfer rather than follow the chain of command. It is also vital for the referring team to make early preparations, alert the necessary staff and ambulance to be on standby when there is a probable need, and wait for the definitive plan from the neurosurgical team before making any arrangements.

When patients with TBI are taken over for immediate surgery, the anaesthetic colleague in the OT is informed regarding the transfer. After patients from peripheral hospitals arrive at HSAJB, they are first reviewed in the ED. The neurosurgical team then reassesses patients and obtains consent from them or their next of kin before posting the case. Despite early alerting of the OT, there is still a prolonged delay from the time of booking to arrival in the OT (median = 80 min) for patients transferred from hospitals with CT scanners to HSAJB. However, this compares favourably to patients directly admitted to HSAJB, wherein the median booking-to-OT time was > 100 min, which could be attributed to pre-notification. Despite this, we realised that the overall time spent in the ED at HSAJB by patients transferred from other hospitals with the intent for immediate surgery was > 2 h (median = 131 min). One simple intervention to overcome this delay would be to transfer patients who require immediate surgery and who remain stable (haemodynamically and neurologically) throughout the transportation directly to the OT, bypassing the ED.

Among the patients directly admitted to our centre, there were delays in acquiring brain CT films and in referring the cases to the neurosurgical team. The time taken to refer the cases to the on-call neurosurgical team after CT ranged from 20 min to 72 min (median = 24 min). Since HSAJB is not a computerised hospital, CT brain films had to be printed before being reviewed by the ED team. The overall median time for the neurosurgical team to review and book cases was 20 min. In 12 cases (7.8%), it took more than an hour to decide on surgery owing to the need for repeated scan or reassessment after withdrawal of sedation.

In level 1 trauma centres, the average CT-to-OT time varies from 68.6 min to 110 min (17, 19). In our study, the minimum CT-to-OT time was 203 min, while the median time was 476 min. We acknowledge that there is no dedicated emergency OT to manage neurotrauma cases. Our team often finds itself competing with other disciplines in the limited resource setting. According to the guidelines on prioritisation of cases for elective and emergency surgeries by the National Perioperative Mortality Review Committee, a life-threatening condition that requires immediate operation without losing patient life or limb is considered an ‘acute emergency’ (27). In the event that an emergency OT is occupied by another case, a second OT has to be mobilised by the anaesthetic team to manage acute emergency cases. Meanwhile, ‘emergency’ is defined as the condition in which life is threatened or morbidity is increased if surgery is not performed within 6 h. With this definition, most traumatic extradural and subdural haemorrhage cases will fall into these two categories, as the risk of increased morbidity and mortality has been well established in the literature. However, only seven of our patients managed to arrive in the OT within 6 h of arrival in the ED. Therefore, we believe that there is a significant room for improvement in this area, and priority access to the OT should be provided for neurosurgery.

The time from arrival in the OT to the start of surgery was 35 min in a study conducted in Canada (17). In our study, the median time was about an hour (62 min), almost twice longer than the previously reported time. While there were three instances in which patients were subjected to exploratory laparotomy leading to extended delays, most other delays prior to initiation of surgery were often attributed to anaesthetic procedures. It is suggested that while the anaesthetic team is establishing monitoring and inserting intravenous lines, the neurosurgical team should prepare the patients’ head (17). We believe that the neurosurgical team should collaborate with the anaesthetic team concurrently rather than consecutively once patients arrive in the OT to shorten the delay.

We also encountered delays related to unique problems not usually seen in other developed nations. In Malaysia, explicit waiver of informed consent is not given even during a medical emergency unless there is no relative or legal guardian available or contactable during the critical period (28). As such, efforts are made to identify the next of kin of patients, counsel them and obtain a meaningful consent. In the United Kingdom, the concept of medical next of kin does not exist. In the event that patients do not have the capacity to make a decision, treatment may be provided by the clinician in the best interest of patients (29). Elsewhere in Europe, when there is a life-threatening emergency requiring urgent intervention, and patients are unfit to provide consent, the attending clinician may institute treatment keeping the best interest of patients in mind (30). However, owing to cultural differences, counselling and consent of family members are necessary prior to surgery in Malaysia.

There is overwhelming evidence to show that patients cared for in specialised trauma or neurotrauma centres fare better than do patients treated in other centres (30, 31). However, a delicate balance is needed in deciding which patient will need specialised neurotrauma care to prevent overcrowding at the neurosurgical centre, which might drain resources and hamper treatment for patients who truly need them. Therefore, it is essential to devise local protocols to ensure optimal use of available resources. An organised trauma system is the key to rapid transportation and timely intervention, which should then be established.

## Conclusion

The door-to-skin time is directly proportional to poor outcomes in patients with TBI. There are still significant delays in patient management leading to delayed surgical interventions at HSAJB, despite being the first regional neurosurgical and trauma referral centre in Malaysia. Concerted efforts from all parties involved in trauma care with established neurotrauma protocols are essential in eliminating such delays.

### Limitations

The main limitation of this study is that the impact of prolonged delays in patient management might have been underestimated, as some patients transferred to our centre for surgical intervention were deemed unsalvageable based on the neurosurgery review. These patients usually included those who experienced extreme delays, but since they were not subjected to surgery, they were excluded from this study. Despite significant delays in the time intervals, no specific reasons were documented in the medical records in many cases. Another limitation of this study is that it was conducted in a single centre. Although most other regional neurosurgery centres in Malaysia have similar setups and referral patterns, the current results cannot be generalised, as there were apparent variations in the geographical profile and population size as well as the availability of resources in other centres. Hence, setting up a nationwide neurotrauma database would be vital and enable future multicentre research to compare performances and outcomes across institutions.

## Figures and Tables

**Table 1:** Baseline demographic and clinical characteristics of patients (*N* = 154)

Characteristics	*n* (%)
Sex	
Male	130 (84.4)
Female	24 (15.6)
Age (years old)^a^	29.65 ± 15.26
Race	
Malay	99 (64.3)
Chinese	21 (13.6)
Indian	18 (11.7)
Others	16 (10.4)
Mechanism	
MVA	123 (79.9)
Fall	25 (16.2)
Assault	3 (1.9)
Others	3 (1.9)
Polytrauma	
No	101 (65.6)
Yes	53 (34.4)
Ventilation	
No	58 (37.7)
Yes	96 (62.3)
Hypotensive episode	
No	139 (83.8)
Yes	25 (16.2)
Deterioration since arrival in the ED	
No	80 (51.9)
Yes	74 (48.1)
Classification of TBI	
Mild	65 (42.2)
Moderate	30 (19.5)
Severe	59 (38.3)
CT findings	
Extradural haemorrhage	73 (47.4)
Subdural haemorrhage	67 (43.5)
Intraparenchymal haemorrhage	10 (6.5)
Multiple intracranial bleeds	4 (2.6)
Surgery	
Craniotomy and clot evacuation	56 (36.4)
Decompressive craniectomy and/or clot evacuation	98 (63.6)

Notes:

a Results are expressed as means ± standard deviations;

MVA = motor vehicle accident; ED = emergency department; TBI = traumatic brain injury; CT = computed tomography

**Table 2:** Time intervals

Times	Direct admission at HSAJB (*n* = 28)	Hospital without CT scanners (*n* = 24)	Hospital with CT scanners (*n* = 97)	Dual transfer (*n* = 5)	Total (*N* = 154)
Door to CT	149 (121–206)	267 (226–302)	103 (226–302)	416 (302–452)	131 (83–233)
CT to neurosurgery review	106 (77–124)	53 (35–72)	383 (272–458)	363 (333–372)	274 (105–413)
Neurosurgery review to booking	30 (20–40)	20 (15–43)	20 (15–32)	20 (12–35)	20 (15–35)
Booking to OT	101 (71–154)	87 (60–117)	80 (20–335)	138 (104–324)	90 (61–130)
OT to skin	59 (40–74)	60 (51–82)	80 (58–118)	138 (104–324)	62 (50–75)
Door to skin	459 (415–511)	520 (300–850)	870 (560–827)	1040 (1020–1155)	605 (494–766)

Note: Results are presented as medians (interquartile ranges) in minutes

**Table 3:** Factors affecting the door-to-skin time

Variables	n	Median (min)	χ^2^	dF	*P* a
Referral pattern	154	605	45.39	3	< 0.001
Direct HSAJB admission	28	459			
Hospital without CT scanners	24	520.0			
Hospital with CT scanners	97	670			
Dual transfer	5	1040			
Severity of TBI			0.689	2	0.709
Mild	65	588			
Moderate	30	668			
Severe	59	600			
Brain CT findings			4.844	3	0.184
Extradural haemorrhage	73	573			
Subdural haemorrhage	57	620			
Intraparenchymal bleed	10	771			
Multiple/DAI	4	599			
Polytrauma			8.314	1	0.007
No	101	579			
Yes	53	679			
Ventilation			0.443	1	0.618
No	58	610			
Yes	96	590			
Hypotensive episode			8.07	1	0.009
No	139	582			
Yes	25	695			

Notes:

a Mood’s median test; significant at *P* < 0.05;

CT = computed tomography; TBI = traumatic brain injury; DAI = diffuse axonal injury

**Table 4:** Clinical factors associated with poor outcomes at discharge and follow-up

Variables	Discharge (*N* = 154)			Follow-up (*N* = 154)		
	
	Good outcome *n* (%)	Poor outcome *n* (%)	*P* a	Good outcome *n* (%)	Poor outcome *n* (%)	*P* a
Polytrauma			0.004			
No	40 (39.6)	61 (60.4)		72 (71.3)	29 (28.7)	0.002
Yes	9 (17.0)	44 (83.0)		24 (45.3)	29 (54.7)	
Hypotensive episode			0.020			
No	46 (35.7)	83 (64.3)		90 (69.8)	39 (30.2)	< 0.001
Yes	3 (12.0)	22 (88.0)		6 (24.0)	19 (76.0)	
Ventilation			< 0.001			
No	35 (60.3)	23 (39.7)		44 (75.9)	14 (24.1)	0.007
Yes	14 (14.6)	82 (85.4)		52 (54.2)	44 (45.8)	
Severe TBI						
No	43 (45.3)	52 (54.7)	< 0.001	68 (71.6)	27 (28.4)	0.003
Yes	6 (10.2)	53 (89.8)		28 (47.5)	31 (52.5)	

Notes:

a Pearson’s chi-squared test; significant at *P* < 0.05;

TBI = traumatic brain injury

**Table 5:** Factors associated with poor outcomes at discharge and follow-up

Factors	Discharge (*N* = 154)		Follow-up (*N* = 154)	
	
	COR^a^ (95% CI)	AOR^b^ (95% CI)	COR^a^ (95% CI)	AOR^b^ (95% CI)
Polytrauma				
Yes	3.21 (1.41, 7.29)*	1.34 (0.50, 3.58)	3.00 (1.50, 5.99)*	1.38 (0.57, 3.37)
No	Reference	Reference	Reference	Reference
Hypotensive episode				
Yes	4.06 (1.15, 14.31)*	0.57 (0.12, 2.69)	7.30 (2.71, 19.70)*	2.43 (0.69, 8.66)
No	Reference	Reference	Reference	Reference
Ventilation				
Yes	8.91 (4.11, 19.31)*	8.12 (2.68, 24.59)*	2.66 (1.29, 5.48)*	2.36 (0.75, 7.46)
No	Reference	Reference	Reference	Reference
Severe TBI				
Yes	7.30 (2.87, 18.62)*	3.04 (0.87, 10.68)	2.79 (1.42, 5.49)*	2.67 (0.86, 8.24)
No	Reference	Reference	Reference	Reference
Door-to-skin time	1.003 (1.000, 1.005)*	1.005 (1.002, 1.008)*	1.007 (1.004, 1.009)*	1.008 (1.005, 1.011)*

Notes:

a Univariable logistic regression using the enter method;

b Multivariable logistic regression using the enter method;

* Significant at *P* < 0.05;

COR = crude odds ratio; AOR = adjusted odds ratio; TBI = traumatic brain injury; This model fits with a Hosmer and Lemeshow *P*-value of 0.633 and Nagelkerke *R*^2^ value of 0.424
